# Structure of the five-coordinate Co^II^ complex (1*H*-imidazole){tris­[(1-benzyl­triazol-4-yl-κ*N* ^3^)meth­yl]amine-κ*N*}cobalt(II) bis­(tetra­fluoro­borate)

**DOI:** 10.1107/S205698902400330X

**Published:** 2024-04-18

**Authors:** Vipul Batra, Garrett C. Reed, David L. Tierney

**Affiliations:** a Department of Chemistry and Biochemistry, Miami University, 651 E. High St., Oxford, Ohio 45056, USA; Texas A & M University, USA

**Keywords:** cobalt(II), five-coordinate, tbta, imidazole, crystal structure

## Abstract

The title compound, [Co(C_3_H_4_N_2_)(C_30_H_30_N_10_)](BF_4_)_2_, is a five-coordinate Co^II^ complex based on the neutral ligands tris­[(1-benzyl­triazol-4-yl)meth­yl]amine (tbta) and imidazole. It exhibits a distorted trigonal bipyramidal geometry in which the equatorial positions are occupied by the three N-atom donors from the triazole rings of the tripodal tbta ligand. The apical amine N-atom donor of tbta and the N-atom donor of the imidazole ligand occupy the axial positions of the coordination sphere. Two tetra­fluoro­borate anions provide charge balance in the crystal.

## Abstract 2

A novel five-coordinate Co^II^ complex based on two neutral ligands {tris­[(1-benzyl­triazol-4-yl)meth­yl]amine and imidazole} demonstrating a distorted trigonal bipyramidal geometry is presented.

## 1. Chemical context

Five-coordinate complexes of Co^II^ are under intense investigation as potential single ion magnets, owing to unusually large magnetic anisotropy. The novel five-coordinate Co^II^ title complex is expected to exhibit similar axial magnetic anisotropy, as it shares a similar geometry with related complexes of tris­[(1-benzyl­triazol-4-yl)meth­yl]amine (tbta) (Mondal *et al.*, 2017 ▸; Schweinfurth *et al.*, 2015 ▸, 2017 ▸), which have shown promising slow magnetic relaxation. This complex pairs two neutral N-atom donor ligands with Co^II^. Notably, the title complex, [Co(imidazole)(tbta)](BF_4_)_2_, represents the first of its kind with a neutral fifth donor, expanding the scope of potential applications within this structural motif. 
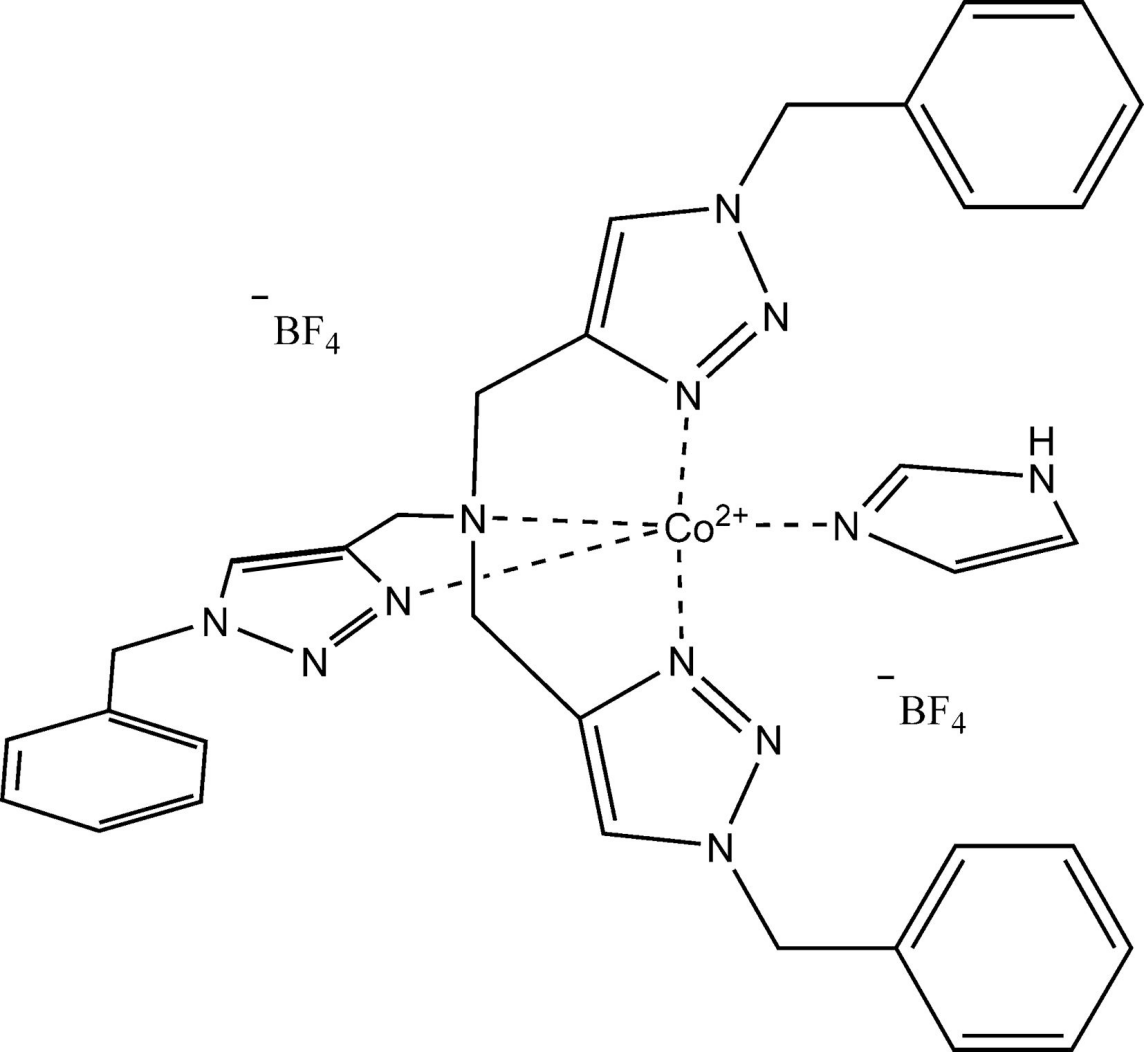


## 2. Structural commentary

**Figure 1:**
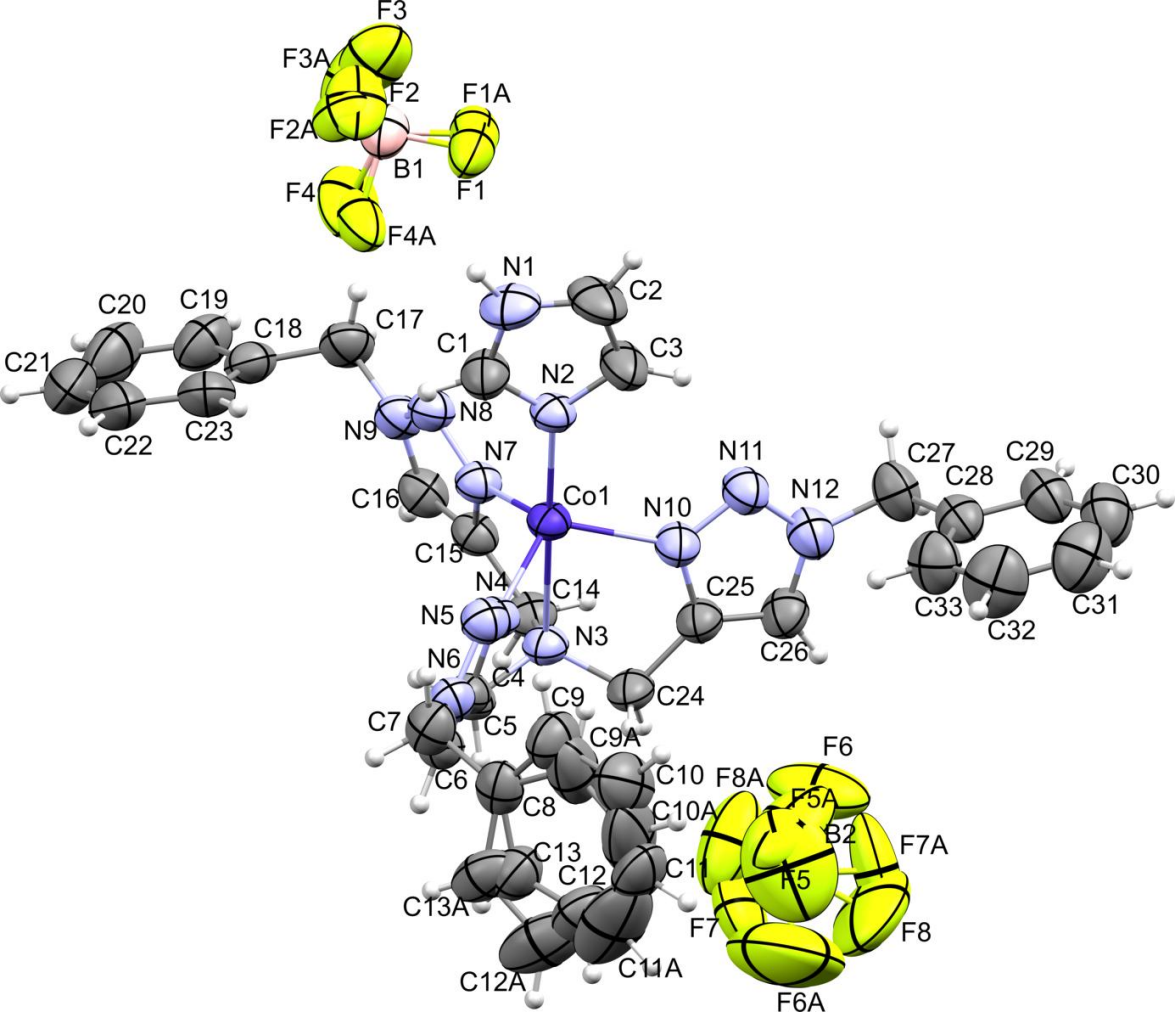
The mol­ecular structure of the title compound. Displacement ellipsoids are drawn at the 50% probability level.

**Figure 2:**
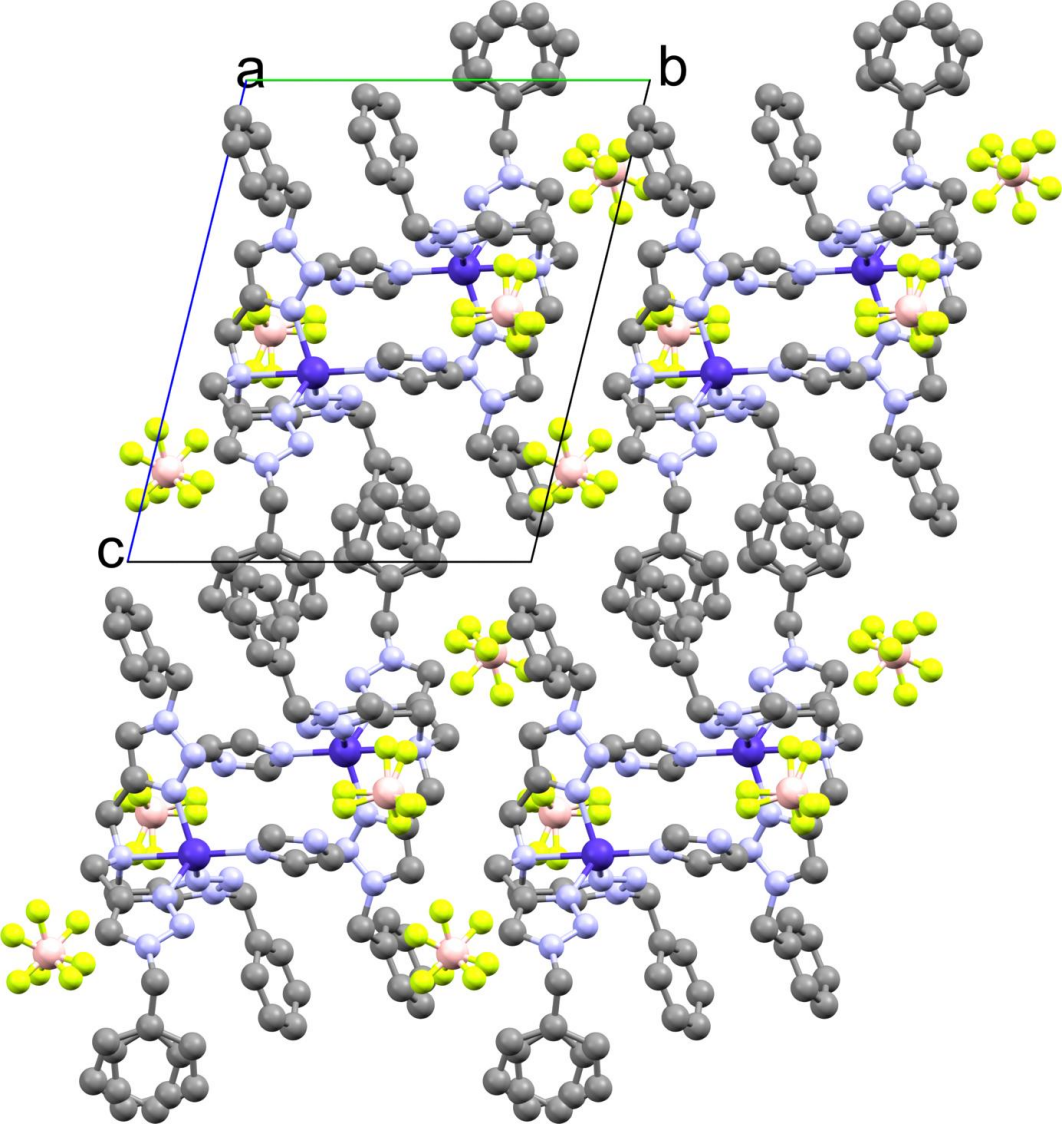
The crystal packing of the title compound. H atoms have been omitted for clarity.

The central metal ion coordinates five N-atom donors, four from the tbta ligand and one from imidazole (Fig. 1 ▸). The Co atom sits 0.51 Å above the equatorial plane (N4/N7/N10) generated by the triazole units of tbta, while the apical N-atom donors form an angle of 178.95 (6)° with respect to the cobalt ion. The geometry about the cobalt center is distorted trigonal bipyramidal (τ_5_ = 1.03; Addison *et al.*, 1984 ▸). A complete list of angles in the coordination sphere is given in Table 1 ▸. Equatorial N-atom donors are present at an average distance of 2.04 Å from the metal ion, and the imidazole N-atom donor is at 2.02 Å. The apical amine N atom of tbta is found at 2.34 Å from the central metal (Table 2 ▸). Two tetra­fluoro­borate counter-ions balance the charge on the metal ion. Both counter-ions, and one of the terminal arene rings, are disordered. The terminal benzyl groups of the tbta ligand, rather than packing upright to form a pocket around the imidazole, are rotated away (Fig. 2 ▸). Two are nearly coplanar at angles of 19.18 (C18–C23) and 15.92° (C28–C33) with respect to the trigonal plane, while the third (C8–C13) is almost normal at an angle of 72.57°. The counter-ions pack nearly along the axial direction of the trigonal bipyramid, where one appears hydrogen bonded to the imidazole N—H group (∼2.2 Å N—H⋯F). The second is translated to a position directly opposite the imidazole, appearing to be shared between two complex mol­ecules.

## 3. Supra­molecular features

The packing of the tbta terminal benzyl groups, as noted above, facilitates the stacking of complexes seen in the extended structure. The complexes pack anti­parallel, with the imidazoles of adjacent complexes approximately coplanar and 4.1 Å apart. The counter-ion hydrogen bonded to the imidazole N—H group appears to be tightly associated with one complex. In contrast, the other counter-ion occupies a position that suggests it is shared between two unit cells. This counter-ion exhibits significantly more disorder than the other, owing to its placement in the lattice. No inter­molecular hydrogen bonding is observed in the extended structure.

## 4. Database survey

The title compound marks the seventh Co^II^ complex with tbta and an ancillary ligand that presents a distorted five-coordinated structure. It is the first with a neutral ancillary ligand, requiring two counter-anions. The neutral imidazole ligand occupies a position closer to the Co^II^ ion, more like the thio­cyanate and azide complexes. The equatorial triazole N-atom donors are remarkably similar across the entire set of compounds. Meanwhile, the apical Co—N distance shows some small variation, trending longer when *trans* to an anionic N-atom donor. This distance in the parent mol­ecule is uniquely short among ancillary N-atom donors in Table 2 ▸.

## 5. Synthesis and crystallization

The click-derived tbta ligand was synthesized according to the literature (Mondal *et al.*, 2017 ▸). The title complex was formed under an inert atmosphere by first preparing a solution of 0.1 mmol tbta and 0.14 mmol imidazole in 10 ml of degassed aceto­nitrile, then adding 0.1 mmol of solid CoBF_4_·6H_2_O. The mixture was stirred for 2 h at room temperature. The solvent was removed under vacuum to reveal a dark-blue crude product. The methanol-soluble fraction produced brown block-shaped crystals by slow evaporation over a period of 2 d.

## 6. Refinement

Crystal data, data collection and structure refinement details are summarized in Table 3 ▸. The H atoms were positioned geometrically (*sp* ^2^-C—H = 0.93 Å, *sp* ^3^-C—H = 0.97 Å and N—H = 0.86 Å) and were refined using a riding model, with *U*_iso_(H) = 1.2*U*_eq_(C) for CH_2_ and C—H hydrogens, and 1.5*U*_eq_(N) for N—H hydrogens.

## Supplementary Material

Crystal structure: contains datablock(s) I, global. DOI: 10.1107/S205698902400330X/jy2045sup1.cif

Structure factors: contains datablock(s) I. DOI: 10.1107/S205698902400330X/jy2045Isup2.hkl

Supporting information file. DOI: 10.1107/S205698902400330X/jy2045Isup4.cml

CCDC reference: 2348506

Additional supporting information:  crystallographic information; 3D view; checkCIF report

## Figures and Tables

**Table 1:** Selected bond angles (°)

N2—Co1—N7	103.73 (6)	N4—Co1—N10	112.62 (7)
N2—Co1—N4	105.56 (6)	N2—Co1—N3	178.95 (6)
N7—Co1—N4	117.04 (6)	N7—Co1—N3	75.67 (6)
N2—Co1—N10	104.25 (6)	N4—Co1—N3	75.50 (6)
N7—Co1—N10	112.12 (7)	N10—Co1—N3	75.26 (6)

**Table 2:** Structural parameters for five-coordinate Co^II^ complexes based on the tbta ligand (distances in Å)

Compound	Co—N_eq_(tbta)	Co—N_ax_(tbta)	Co—*X*_ax_	Reference	CSD refcode
[Co(tbta)(Im)](BF_4_)_2_	2.04	2.34	2.02 (N)	This work	This work
[Co(tbta)(N_3_)]ClO_4_·3CH_3_CN	2.04	2.37	1.96 (N)	Schweinfurth *et al.* (2015 ▸)	RUDDUR
[Co(tbta)(NCS)]BF_4_	2.03	2.37	1.98 (N)	Schweinfurth *et al.* (2017 ▸)	HAWYOW
[Co(tbta)Cl]BF_4_	2.04	2.39	2.26 (Cl)	Schweinfurth *et al.* (2017 ▸)	HAWXEL
[Co(tbta)(NCS)]BF_4_·3CH_3_CN	2.03	2.35	1.95 (N)	Schweinfurth *et al.* (2017 ▸)	HAWXAH
[Co(tbta)(Br)]ClO_4_	2.05	2.33	2.40 (Br)	Mondal *et al.* (2017 ▸)	KENWUY
[Co(tbta)(Cl)]ClO_4_·2CH_3_CN·H_2_O	2.04	2.34	2.26 (Cl)	Mondal *et al.* (2017 ▸)	KENWOS

**Table 3:** Experimental details

Crystal data	
Chemical formula	[Co(C_3_H_4_N_2_)(C_30_H_30_N_10_)](BF_4_)_2_
*M*_r_	831.27
Crystal system, space group	Triclinic, *P* 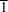
Temperature (K)	297
*a*, *b*, *c* (Å)	10.6861 (4), 13.0639 (5), 15.7006 (6)
α, β, γ (°)	96.304 (2), 107.142 (2), 110.766 (2)
*V* (Å^3^)	1901.24 (13)
*Z*	2
Radiation type	Mo *K*α
μ (mm^−1^)	0.53
Crystal size (mm)	0.23 × 0.16 × 0.13

Data collection	
Diffractometer	Bruker APEXII CCD
Absorption correction	Multi-scan (*SADABS*; Bruker, 2016 ▸; Krause *et al.*, 2015 ▸)
*T*_min_, *T*_max_	0.681, 0.746
No. of measured, independent and observed [*I* > 2σ(*I*)] reflections	212950, 11085, 8422
*R*_int_	0.053
(sin θ/λ)_max_ (Å^−1^)	0.705

Refinement	
*R*[*F* ^2^ > 2σ(*F* ^2^)], *wR*(*F* ^2^), *S*	0.049, 0.118, 1.04
No. of reflections	11085
No. of parameters	622
No. of restraints	19
H-atom treatment	H-atom parameters constrained
Δρ_max_, Δρ_min_ (e Å^−3^)	0.30, −0.31

Computer programs: *APEX3* (Bruker, 2016 ▸), *SAINT* (Bruker, 2016 ▸), *SHELXT2018* (Sheldrick, 2015*a* ▸), *SHELXL2019* (Sheldrick, 2015*b* ▸), *publCIF* (Westrip, 2010 ▸) and *Mercury* (Macrae *et al.*, 2020 ▸).

## supplementary crystallographic information

### Crystal data

**Table:**

[Co(C_3_H_4_N_2_)(C_30_H_30_N_10_)](BF_4_)_2_	*Z* = 2
*M**_r_* = 831.27	*F*(000) = 850
Triclinic, *P*1	*D*_x_ = 1.452 Mg m^−^^3^
*a* = 10.6861 (4) Å	Mo *K*α radiation, λ = 0.71073 Å
*b* = 13.0639 (5) Å	Cell parameters from 9079 reflections
*c* = 15.7006 (6) Å	θ = 2.8–29.5°
α = 96.304 (2)°	µ = 0.53 mm^−^^1^
β = 107.142 (2)°	*T* = 297 K
γ = 110.766 (2)°	Block, brown
*V* = 1901.24 (13) Å^3^	0.23 × 0.16 × 0.13 mm

### Data collection

**Table:**

Bruker APEXII CCD diffractometer	8422 reflections with *I* > 2σ(*I*)
Detector resolution: 8.33 pixels mm^-1^	*R*_int_ = 0.053
φ and ω scans	θ_max_ = 30.1°, θ_min_ = 2.1°
Absorption correction: multi-scan (SADABS; Bruker, 2016; Krause *et al.*, 2015)	*h* = −15→15
*T*_min_ = 0.681, *T*_max_ = 0.746	*k* = −18→18
212950 measured reflections	*l* = −22→22
11085 independent reflections	

### Refinement

**Table:**

Refinement on *F*^2^	Primary atom site location: dual
Least-squares matrix: full	Secondary atom site location: difference Fourier map
*R*[*F*^2^ > 2σ(*F*^2^)] = 0.049	Hydrogen site location: inferred from neighbouring sites
*wR*(*F*^2^) = 0.118	H-atom parameters constrained
*S* = 1.04	*w* = 1/[σ^2^(*F*_o_^2^) + (0.0411*P*)^2^ + 0.8855*P*] where *P* = (*F*_o_^2^ + 2*F*_c_^2^)/3
11085 reflections	(Δ/σ)_max_ = 0.001
622 parameters	Δρ_max_ = 0.30 e Å^−^^3^
19 restraints	Δρ_min_ = −0.31 e Å^−^^3^

### Special details

**Table:**

Geometry. All e.s.d.'s (except the e.s.d. in the dihedral angle between two l.s. planes) are estimated using the full covariance matrix. The cell e.s.d.'s are taken into account individually in the estimation of e.s.d.'s in distances, angles and torsion angles; correlations between e.s.d.'s in cell parameters are only used when they are defined by crystal symmetry. An approximate (isotropic) treatment of cell e.s.d.'s is used for estimating e.s.d.'s involving l.s. planes.

### Fractional atomic coordinates and isotropic or equivalent isotropic displacement parameters (Å^2^)

**Table:**

	*x*	*y*	*z*	*U*_iso_*/*U*_eq_	Occ. (<1)
Co1	0.36020 (3)	0.34470 (2)	0.60765 (2)	0.04356 (8)	
F1A	0.2525 (11)	0.8281 (18)	0.5171 (11)	0.099 (5)	0.5
F1	0.2441 (11)	0.8323 (18)	0.5417 (11)	0.082 (3)	0.5
F2	0.0306 (12)	0.8431 (8)	0.5049 (6)	0.078 (2)	0.5
F2A	0.0540 (13)	0.8592 (8)	0.5050 (8)	0.100 (3)	0.5
F3	0.1035 (14)	0.8054 (8)	0.3951 (5)	0.114 (3)	0.5
F3A	0.0676 (14)	0.7550 (8)	0.3854 (6)	0.122 (4)	0.5
F4	0.0432 (12)	0.6776 (7)	0.4778 (10)	0.122 (4)	0.5
F4A	0.0559 (9)	0.6868 (6)	0.5085 (10)	0.104 (3)	0.5
F5	0.7255 (15)	0.1135 (11)	0.8639 (8)	0.196 (6)	0.5
F5A	0.7325 (14)	0.1367 (9)	0.8346 (6)	0.164 (5)	0.5
F6	0.7922 (10)	0.0967 (5)	0.7480 (5)	0.149 (2)	0.5
F6A	0.7169 (16)	−0.0137 (12)	0.8717 (10)	0.280 (6)	0.5
F7	0.6539 (5)	−0.0566 (4)	0.7751 (6)	0.1246 (19)	0.5
F7A	0.8787 (5)	0.0559 (6)	0.8182 (5)	0.148 (4)	0.5
F8	0.8653 (9)	0.0314 (8)	0.8542 (8)	0.199 (5)	0.5
F8A	0.6436 (7)	−0.0088 (7)	0.7250 (5)	0.152 (3)	0.5
N1	0.3017 (2)	0.63686 (16)	0.57980 (15)	0.0699 (5)	
H1	0.246508	0.671656	0.565345	0.084*	
N2	0.36964 (17)	0.50047 (12)	0.60048 (11)	0.0460 (3)	
N3	0.35140 (17)	0.16428 (12)	0.61403 (11)	0.0475 (3)	
N4	0.25689 (17)	0.29742 (13)	0.69646 (11)	0.0488 (4)	
N5	0.22898 (18)	0.35746 (14)	0.75569 (12)	0.0534 (4)	
N6	0.16517 (18)	0.28550 (15)	0.79868 (11)	0.0545 (4)	
N7	0.25982 (17)	0.25491 (12)	0.47541 (11)	0.0467 (3)	
N8	0.19169 (18)	0.28138 (13)	0.40209 (11)	0.0503 (4)	
N9	0.14603 (19)	0.19319 (14)	0.33275 (11)	0.0540 (4)	
N10	0.57001 (18)	0.36719 (13)	0.65806 (13)	0.0540 (4)	
N11	0.68556 (19)	0.45018 (15)	0.65897 (13)	0.0591 (4)	
N12	0.79108 (19)	0.41538 (15)	0.68478 (14)	0.0611 (4)	
C1	0.2593 (3)	0.52657 (18)	0.57280 (17)	0.0638 (6)	
H1A	0.164029	0.474950	0.551155	0.077*	
C2	0.4445 (3)	0.68414 (19)	0.6131 (2)	0.0781 (7)	
H2	0.503178	0.760336	0.625526	0.094*	
C3	0.4865 (3)	0.59987 (18)	0.6251 (2)	0.0724 (7)	
H3	0.581285	0.608100	0.646940	0.087*	
C4	0.2231 (2)	0.10509 (15)	0.63579 (14)	0.0519 (4)	
H4A	0.232463	0.043631	0.662777	0.062*	
H4B	0.138308	0.074121	0.580297	0.062*	
C5	0.2102 (2)	0.18828 (15)	0.70182 (13)	0.0471 (4)	
C6	0.1520 (2)	0.18019 (17)	0.76808 (14)	0.0546 (5)	
H6	0.111784	0.116138	0.788033	0.066*	
C7	0.1203 (3)	0.3250 (2)	0.87159 (16)	0.0655 (6)	
H7A	0.025695	0.270839	0.864156	0.079*	
H7B	0.114029	0.396273	0.865501	0.079*	
C8	0.2227 (2)	0.3399 (2)	0.96547 (15)	0.0622 (5)	
C9	0.3088 (13)	0.4565 (8)	1.0052 (8)	0.079 (2)	0.5
H9	0.294336	0.510554	0.974382	0.095*	0.5
C9A	0.3382 (13)	0.4315 (10)	1.0210 (8)	0.110 (5)	0.5
H9A	0.363220	0.498509	1.002057	0.132*	0.5
C10A	0.4208 (15)	0.4275 (15)	1.1064 (9)	0.142 (8)	0.5
H10A	0.496406	0.492544	1.146208	0.170*	0.5
C10	0.4156 (12)	0.4881 (7)	1.0919 (7)	0.095 (3)	0.5
H10	0.474392	0.563721	1.119591	0.114*	0.5
C11	0.4331 (15)	0.4037 (13)	1.1366 (7)	0.092 (3)	0.5
H11	0.506230	0.422843	1.193140	0.111*	0.5
C11A	0.389 (2)	0.3236 (19)	1.1320 (9)	0.169 (12)	0.5
H11A	0.446306	0.319685	1.187934	0.203*	0.5
C12	0.3450 (17)	0.2972 (12)	1.0976 (8)	0.096 (3)	0.5
H12	0.352928	0.243195	1.130306	0.115*	0.5
C12A	0.2786 (15)	0.2324 (12)	1.0776 (7)	0.127 (4)	0.5
H12A	0.259872	0.163460	1.093508	0.152*	0.5
C13A	0.1901 (13)	0.2404 (10)	0.9958 (8)	0.083 (3)	0.5
H13A	0.106780	0.177653	0.960329	0.099*	0.5
C13	0.2427 (11)	0.2624 (11)	1.0117 (9)	0.079 (3)	0.5
H13	0.187312	0.185938	0.985220	0.095*	0.5
C14	0.3398 (2)	0.10908 (16)	0.52329 (15)	0.0553 (5)	
H14A	0.291509	0.027895	0.512534	0.066*	
H14B	0.434590	0.127033	0.520738	0.066*	
C15	0.2564 (2)	0.15038 (15)	0.45181 (14)	0.0486 (4)	
C16	0.1827 (2)	0.11045 (17)	0.36068 (15)	0.0564 (5)	
H16	0.161605	0.040725	0.324798	0.068*	
C17	0.0726 (3)	0.1974 (2)	0.23878 (15)	0.0658 (6)	
H17A	0.112458	0.169520	0.198112	0.079*	
H17B	0.091285	0.275276	0.237201	0.079*	
C18	−0.0860 (3)	0.12992 (19)	0.20364 (15)	0.0610 (5)	
C19	−0.1537 (3)	0.0617 (2)	0.11573 (18)	0.0823 (8)	
H19	−0.100147	0.055110	0.079913	0.099*	
C20	−0.3023 (4)	0.0027 (3)	0.0807 (3)	0.1080 (12)	
H20	−0.347858	−0.044001	0.021787	0.130*	
C21	−0.3810 (4)	0.0134 (3)	0.1332 (3)	0.1083 (12)	
H21	−0.480250	−0.025614	0.109682	0.130*	
C22	−0.3143 (3)	0.0814 (3)	0.2200 (3)	0.0934 (9)	
H22	−0.368230	0.088724	0.255385	0.112*	
C23	−0.1678 (3)	0.1390 (2)	0.25514 (18)	0.0739 (7)	
H23	−0.123164	0.184765	0.314428	0.089*	
C24	0.4849 (2)	0.17805 (17)	0.68650 (16)	0.0571 (5)	
H24A	0.505512	0.112223	0.675713	0.069*	
H24B	0.475498	0.187227	0.746142	0.069*	
C25	0.6027 (2)	0.28067 (17)	0.68347 (15)	0.0541 (5)	
C26	0.7445 (2)	0.31154 (19)	0.70004 (17)	0.0621 (5)	
H26	0.798057	0.269821	0.718105	0.075*	
C27	0.9359 (2)	0.4905 (2)	0.69431 (18)	0.0702 (6)	
H27A	0.929410	0.542044	0.654856	0.084*	
H27B	0.981539	0.445886	0.673334	0.084*	
C28	1.0287 (2)	0.55804 (17)	0.79105 (16)	0.0575 (5)	
C29	1.1738 (3)	0.6157 (2)	0.8106 (2)	0.0705 (6)	
H29	1.211690	0.608547	0.765086	0.085*	
C30	1.2629 (3)	0.6833 (2)	0.8963 (2)	0.0869 (8)	
H30	1.360261	0.722037	0.908040	0.104*	
C31	1.2105 (4)	0.6941 (3)	0.9638 (2)	0.0971 (9)	
H31	1.271301	0.740199	1.021701	0.117*	
C32	1.0672 (4)	0.6367 (3)	0.9461 (2)	0.0993 (10)	
H32	1.030809	0.643570	0.992346	0.119*	
C33	0.9763 (3)	0.5687 (2)	0.86031 (19)	0.0775 (7)	
H33	0.879207	0.529931	0.849173	0.093*	
B1	0.1061 (3)	0.7874 (2)	0.4793 (2)	0.0643 (6)	
B2	0.7495 (4)	0.0447 (3)	0.8123 (3)	0.0810 (9)	

### Atomic displacement parameters (Å^2^)

**Table:**

	*U*^11^	*U*^22^	*U*^33^	*U*^12^	*U*^13^	*U*^23^
Co1	0.04847 (14)	0.03160 (12)	0.04817 (14)	0.01636 (10)	0.01373 (11)	0.01019 (9)
F1A	0.059 (3)	0.065 (6)	0.151 (11)	0.017 (3)	0.011 (4)	0.044 (7)
F1	0.060 (3)	0.061 (4)	0.101 (5)	0.026 (3)	−0.001 (3)	0.012 (4)
F2	0.085 (4)	0.097 (5)	0.081 (4)	0.061 (4)	0.038 (3)	0.029 (4)
F2A	0.092 (5)	0.049 (2)	0.141 (6)	0.033 (2)	0.025 (3)	−0.013 (3)
F3	0.129 (7)	0.169 (9)	0.074 (4)	0.085 (7)	0.047 (4)	0.034 (4)
F3A	0.111 (6)	0.165 (8)	0.086 (3)	0.083 (6)	0.014 (3)	−0.019 (4)
F4	0.098 (4)	0.040 (2)	0.165 (8)	0.004 (2)	0.002 (4)	−0.008 (3)
F4A	0.069 (4)	0.065 (4)	0.192 (10)	0.031 (3)	0.056 (5)	0.046 (5)
F5	0.222 (9)	0.207 (11)	0.182 (9)	0.094 (7)	0.126 (8)	−0.043 (7)
F5A	0.208 (9)	0.133 (5)	0.144 (6)	0.126 (6)	−0.001 (6)	−0.003 (5)
F6	0.249 (7)	0.102 (3)	0.185 (5)	0.102 (4)	0.145 (6)	0.079 (4)
F6A	0.325 (14)	0.303 (13)	0.350 (15)	0.155 (11)	0.216 (13)	0.249 (13)
F7	0.076 (3)	0.080 (3)	0.167 (6)	−0.008 (2)	0.034 (3)	−0.003 (3)
F7A	0.061 (3)	0.149 (5)	0.163 (6)	−0.013 (3)	0.054 (3)	−0.087 (5)
F8	0.158 (7)	0.203 (7)	0.249 (9)	0.132 (6)	0.010 (6)	0.081 (7)
F8A	0.116 (4)	0.178 (7)	0.137 (5)	0.098 (5)	−0.007 (4)	−0.041 (4)
N1	0.0898 (15)	0.0575 (11)	0.0846 (14)	0.0477 (11)	0.0356 (12)	0.0292 (10)
N2	0.0526 (9)	0.0340 (7)	0.0504 (9)	0.0174 (6)	0.0168 (7)	0.0112 (6)
N3	0.0518 (9)	0.0383 (7)	0.0528 (9)	0.0203 (7)	0.0157 (7)	0.0141 (7)
N4	0.0554 (9)	0.0383 (7)	0.0506 (9)	0.0182 (7)	0.0170 (7)	0.0116 (7)
N5	0.0573 (10)	0.0478 (9)	0.0534 (9)	0.0210 (8)	0.0186 (8)	0.0108 (7)
N6	0.0552 (10)	0.0594 (10)	0.0467 (9)	0.0227 (8)	0.0160 (8)	0.0135 (8)
N7	0.0531 (9)	0.0377 (7)	0.0492 (9)	0.0187 (7)	0.0179 (7)	0.0103 (6)
N8	0.0557 (9)	0.0435 (8)	0.0494 (9)	0.0175 (7)	0.0178 (7)	0.0141 (7)
N9	0.0587 (10)	0.0511 (9)	0.0467 (9)	0.0167 (8)	0.0188 (8)	0.0110 (7)
N10	0.0490 (9)	0.0419 (8)	0.0692 (11)	0.0188 (7)	0.0175 (8)	0.0158 (8)
N11	0.0545 (10)	0.0491 (9)	0.0743 (12)	0.0203 (8)	0.0240 (9)	0.0176 (8)
N12	0.0521 (10)	0.0551 (10)	0.0743 (12)	0.0198 (8)	0.0240 (9)	0.0115 (9)
C1	0.0576 (12)	0.0485 (11)	0.0812 (16)	0.0243 (10)	0.0164 (11)	0.0145 (10)
C2	0.0851 (18)	0.0403 (11)	0.115 (2)	0.0228 (11)	0.0432 (16)	0.0299 (13)
C3	0.0552 (13)	0.0461 (11)	0.113 (2)	0.0168 (10)	0.0270 (13)	0.0283 (12)
C4	0.0559 (11)	0.0369 (9)	0.0573 (11)	0.0162 (8)	0.0149 (9)	0.0147 (8)
C5	0.0469 (10)	0.0397 (9)	0.0479 (10)	0.0143 (8)	0.0109 (8)	0.0127 (8)
C6	0.0548 (11)	0.0504 (11)	0.0525 (11)	0.0174 (9)	0.0142 (9)	0.0169 (9)
C7	0.0643 (13)	0.0801 (15)	0.0593 (13)	0.0352 (12)	0.0256 (11)	0.0146 (11)
C8	0.0553 (12)	0.0833 (16)	0.0509 (12)	0.0270 (11)	0.0256 (10)	0.0130 (11)
C9	0.077 (5)	0.079 (4)	0.062 (5)	0.027 (4)	0.007 (3)	0.008 (3)
C9A	0.074 (6)	0.142 (10)	0.066 (5)	0.000 (6)	0.016 (4)	0.016 (6)
C10A	0.081 (6)	0.192 (19)	0.076 (9)	0.002 (11)	−0.007 (6)	0.023 (10)
C10	0.095 (5)	0.080 (5)	0.070 (5)	0.012 (4)	0.011 (4)	0.002 (4)
C11	0.081 (6)	0.132 (8)	0.055 (6)	0.044 (5)	0.009 (5)	0.031 (5)
C11A	0.15 (2)	0.34 (4)	0.072 (10)	0.14 (2)	0.064 (12)	0.088 (17)
C12	0.109 (9)	0.113 (7)	0.062 (7)	0.046 (6)	0.022 (6)	0.030 (6)
C12A	0.189 (13)	0.204 (13)	0.081 (6)	0.146 (11)	0.082 (8)	0.073 (8)
C13A	0.100 (9)	0.124 (8)	0.065 (5)	0.073 (6)	0.044 (5)	0.044 (5)
C13	0.072 (6)	0.095 (5)	0.070 (5)	0.039 (4)	0.018 (4)	0.026 (4)
C14	0.0653 (12)	0.0400 (9)	0.0653 (13)	0.0267 (9)	0.0236 (10)	0.0117 (9)
C15	0.0540 (11)	0.0382 (9)	0.0546 (11)	0.0178 (8)	0.0226 (9)	0.0090 (8)
C16	0.0630 (12)	0.0462 (10)	0.0570 (12)	0.0187 (9)	0.0242 (10)	0.0049 (9)
C17	0.0747 (15)	0.0703 (14)	0.0475 (11)	0.0226 (12)	0.0216 (11)	0.0199 (10)
C18	0.0710 (14)	0.0577 (12)	0.0524 (12)	0.0266 (11)	0.0154 (10)	0.0228 (10)
C19	0.0873 (19)	0.0848 (18)	0.0636 (15)	0.0403 (15)	0.0070 (14)	0.0125 (13)
C20	0.102 (3)	0.093 (2)	0.085 (2)	0.035 (2)	−0.0170 (19)	0.0059 (17)
C21	0.0706 (19)	0.108 (3)	0.127 (3)	0.0287 (18)	0.007 (2)	0.058 (2)
C22	0.0790 (19)	0.110 (2)	0.110 (3)	0.0450 (18)	0.0376 (18)	0.066 (2)
C23	0.0803 (17)	0.0781 (16)	0.0683 (15)	0.0314 (14)	0.0283 (13)	0.0353 (13)
C24	0.0553 (11)	0.0446 (10)	0.0691 (13)	0.0233 (9)	0.0127 (10)	0.0208 (9)
C25	0.0518 (11)	0.0464 (10)	0.0634 (12)	0.0228 (9)	0.0154 (9)	0.0157 (9)
C26	0.0563 (12)	0.0568 (12)	0.0753 (15)	0.0293 (10)	0.0190 (11)	0.0150 (11)
C27	0.0567 (13)	0.0732 (15)	0.0786 (16)	0.0189 (11)	0.0335 (12)	0.0108 (12)
C28	0.0567 (12)	0.0491 (10)	0.0747 (14)	0.0266 (9)	0.0286 (11)	0.0149 (10)
C29	0.0623 (14)	0.0601 (13)	0.0910 (18)	0.0215 (11)	0.0356 (13)	0.0151 (12)
C30	0.0662 (16)	0.0694 (16)	0.104 (2)	0.0163 (13)	0.0202 (16)	0.0065 (15)
C31	0.096 (2)	0.089 (2)	0.086 (2)	0.0380 (18)	0.0155 (18)	−0.0062 (16)
C32	0.104 (2)	0.126 (3)	0.0771 (19)	0.057 (2)	0.0404 (18)	0.0040 (18)
C33	0.0671 (15)	0.0917 (19)	0.0836 (18)	0.0370 (14)	0.0372 (14)	0.0144 (15)
B1	0.0582 (15)	0.0537 (14)	0.0739 (17)	0.0254 (12)	0.0138 (13)	0.0067 (12)
B2	0.084 (2)	0.079 (2)	0.093 (2)	0.0376 (18)	0.047 (2)	0.0152 (18)

### Geometric parameters (Å, º)

**Table:**

Co1—N2	2.0188 (14)	C8—C13A	1.396 (10)
Co1—N7	2.0242 (16)	C8—C9	1.420 (10)
Co1—N4	2.0390 (16)	C9—C10	1.397 (12)
Co1—N10	2.0433 (17)	C9—H9	0.9300
Co1—N3	2.3398 (15)	C9A—C10A	1.389 (12)
F1A—B1	1.369 (11)	C9A—H9A	0.9300
F1—B1	1.374 (10)	C10A—C11A	1.41 (2)
F2—B1	1.374 (8)	C10A—H10A	0.9300
F2A—B1	1.332 (8)	C10—C11	1.408 (16)
F3—B1	1.362 (8)	C10—H10	0.9300
F3A—B1	1.380 (9)	C11—C12	1.32 (2)
F4—B1	1.344 (9)	C11—H11	0.9300
F4A—B1	1.414 (9)	C11A—C12A	1.31 (2)
F5—B2	1.286 (7)	C11A—H11A	0.9300
F5A—B2	1.303 (8)	C12—C13	1.366 (12)
F6—B2	1.374 (5)	C12—H12	0.9300
F6A—B2	1.317 (7)	C12A—C13A	1.392 (12)
F7—B2	1.288 (5)	C12A—H12A	0.9300
F7A—B2	1.309 (6)	C13A—H13A	0.9300
F8—B2	1.301 (7)	C13—H13	0.9300
F8A—B2	1.401 (6)	C14—C15	1.493 (3)
N1—C1	1.329 (3)	C14—H14A	0.9700
N1—C2	1.332 (3)	C14—H14B	0.9700
N1—H1	0.8600	C15—C16	1.349 (3)
N2—C1	1.308 (3)	C16—H16	0.9300
N2—C3	1.360 (3)	C17—C18	1.495 (3)
N3—C14	1.474 (3)	C17—H17A	0.9700
N3—C4	1.475 (3)	C17—H17B	0.9700
N3—C24	1.476 (3)	C18—C23	1.379 (4)
N4—N5	1.318 (2)	C18—C19	1.381 (3)
N4—C5	1.355 (2)	C19—C20	1.393 (5)
N5—N6	1.327 (2)	C19—H19	0.9300
N6—C6	1.349 (3)	C20—C21	1.369 (5)
N6—C7	1.475 (3)	C20—H20	0.9300
N7—N8	1.322 (2)	C21—C22	1.367 (5)
N7—C15	1.360 (2)	C21—H21	0.9300
N8—N9	1.328 (2)	C22—C23	1.374 (4)
N9—C16	1.348 (3)	C22—H22	0.9300
N9—C17	1.470 (3)	C23—H23	0.9300
N10—N11	1.318 (2)	C24—C25	1.495 (3)
N10—C25	1.362 (2)	C24—H24A	0.9700
N11—N12	1.333 (3)	C24—H24B	0.9700
N12—C26	1.345 (3)	C25—C26	1.355 (3)
N12—C27	1.461 (3)	C26—H26	0.9300
C1—H1A	0.9300	C27—C28	1.505 (3)
C2—C3	1.340 (3)	C27—H27A	0.9700
C2—H2	0.9300	C27—H27B	0.9700
C3—H3	0.9300	C28—C33	1.377 (3)
C4—C5	1.489 (3)	C28—C29	1.381 (3)
C4—H4A	0.9700	C29—C30	1.373 (4)
C4—H4B	0.9700	C29—H29	0.9300
C5—C6	1.356 (3)	C30—C31	1.353 (4)
C6—H6	0.9300	C30—H30	0.9300
C7—C8	1.498 (3)	C31—C32	1.369 (5)
C7—H7A	0.9700	C31—H31	0.9300
C7—H7B	0.9700	C32—C33	1.378 (4)
C8—C9A	1.343 (10)	C32—H32	0.9300
C8—C13	1.349 (10)	C33—H33	0.9300
			
N2—Co1—N7	103.73 (6)	C12A—C13A—H13A	119.0
N2—Co1—N4	105.56 (6)	C8—C13A—H13A	119.0
N7—Co1—N4	117.04 (6)	C8—C13—C12	119.4 (11)
N2—Co1—N10	104.25 (6)	C8—C13—H13	120.3
N7—Co1—N10	112.12 (7)	C12—C13—H13	120.3
N4—Co1—N10	112.62 (7)	N3—C14—C15	108.47 (15)
N2—Co1—N3	178.95 (6)	N3—C14—H14A	110.0
N7—Co1—N3	75.67 (6)	C15—C14—H14A	110.0
N4—Co1—N3	75.50 (6)	N3—C14—H14B	110.0
N10—Co1—N3	75.26 (6)	C15—C14—H14B	110.0
C1—N1—C2	108.14 (19)	H14A—C14—H14B	108.4
C1—N1—H1	125.9	C16—C15—N7	107.32 (18)
C2—N1—H1	125.9	C16—C15—C14	133.89 (18)
C1—N2—C3	105.12 (17)	N7—C15—C14	118.49 (17)
C1—N2—Co1	125.60 (14)	N9—C16—C15	105.28 (18)
C3—N2—Co1	129.24 (14)	N9—C16—H16	127.4
C14—N3—C4	111.60 (16)	C15—C16—H16	127.4
C14—N3—C24	111.48 (16)	N9—C17—C18	113.59 (18)
C4—N3—C24	112.17 (16)	N9—C17—H17A	108.8
C14—N3—Co1	107.63 (11)	C18—C17—H17A	108.8
C4—N3—Co1	106.16 (11)	N9—C17—H17B	108.8
C24—N3—Co1	107.45 (11)	C18—C17—H17B	108.8
N5—N4—C5	110.35 (16)	H17A—C17—H17B	107.7
N5—N4—Co1	130.65 (12)	C23—C18—C19	119.0 (3)
C5—N4—Co1	118.96 (13)	C23—C18—C17	121.5 (2)
N4—N5—N6	105.52 (15)	C19—C18—C17	119.5 (2)
N5—N6—C6	111.96 (17)	C18—C19—C20	120.0 (3)
N5—N6—C7	120.22 (18)	C18—C19—H19	120.0
C6—N6—C7	127.82 (19)	C20—C19—H19	120.0
N8—N7—C15	110.09 (16)	C21—C20—C19	119.9 (3)
N8—N7—Co1	129.88 (12)	C21—C20—H20	120.0
C15—N7—Co1	120.03 (13)	C19—C20—H20	120.0
N7—N8—N9	105.51 (15)	C22—C21—C20	120.2 (3)
N8—N9—C16	111.80 (17)	C22—C21—H21	119.9
N8—N9—C17	119.83 (18)	C20—C21—H21	119.9
C16—N9—C17	128.28 (19)	C21—C22—C23	120.1 (3)
N11—N10—C25	110.20 (17)	C21—C22—H22	119.9
N11—N10—Co1	129.57 (13)	C23—C22—H22	119.9
C25—N10—Co1	119.47 (13)	C22—C23—C18	120.9 (3)
N10—N11—N12	105.55 (16)	C22—C23—H23	119.6
N11—N12—C26	111.82 (18)	C18—C23—H23	119.6
N11—N12—C27	119.04 (19)	N3—C24—C25	107.29 (16)
C26—N12—C27	129.1 (2)	N3—C24—H24A	110.3
N2—C1—N1	110.8 (2)	C25—C24—H24A	110.3
N2—C1—H1A	124.6	N3—C24—H24B	110.3
N1—C1—H1A	124.6	C25—C24—H24B	110.3
N1—C2—C3	106.2 (2)	H24A—C24—H24B	108.5
N1—C2—H2	126.9	C26—C25—N10	107.14 (18)
C3—C2—H2	126.9	C26—C25—C24	134.62 (19)
C2—C3—N2	109.7 (2)	N10—C25—C24	118.23 (18)
C2—C3—H3	125.1	N12—C26—C25	105.27 (19)
N2—C3—H3	125.1	N12—C26—H26	127.4
N3—C4—C5	107.84 (15)	C25—C26—H26	127.4
N3—C4—H4A	110.1	N12—C27—C28	113.7 (2)
C5—C4—H4A	110.1	N12—C27—H27A	108.8
N3—C4—H4B	110.1	C28—C27—H27A	108.8
C5—C4—H4B	110.1	N12—C27—H27B	108.8
H4A—C4—H4B	108.5	C28—C27—H27B	108.8
N4—C5—C6	107.33 (17)	H27A—C27—H27B	107.7
N4—C5—C4	118.52 (17)	C33—C28—C29	118.2 (2)
C6—C5—C4	134.04 (17)	C33—C28—C27	123.7 (2)
N6—C6—C5	104.85 (17)	C29—C28—C27	118.1 (2)
N6—C6—H6	127.6	C30—C29—C28	120.9 (3)
C5—C6—H6	127.6	C30—C29—H29	119.6
N6—C7—C8	111.97 (18)	C28—C29—H29	119.6
N6—C7—H7A	109.2	C31—C30—C29	120.6 (3)
C8—C7—H7A	109.2	C31—C30—H30	119.7
N6—C7—H7B	109.2	C29—C30—H30	119.7
C8—C7—H7B	109.2	C30—C31—C32	119.4 (3)
H7A—C7—H7B	107.9	C30—C31—H31	120.3
C9A—C8—C13A	117.8 (7)	C32—C31—H31	120.3
C13—C8—C9	120.3 (7)	C31—C32—C33	120.7 (3)
C9A—C8—C7	129.9 (5)	C31—C32—H32	119.7
C13—C8—C7	130.2 (5)	C33—C32—H32	119.7
C13A—C8—C7	112.3 (5)	C28—C33—C32	120.3 (3)
C9—C8—C7	109.4 (4)	C28—C33—H33	119.9
C10—C9—C8	118.3 (8)	C32—C33—H33	119.9
C10—C9—H9	120.8	F4—B1—F3	113.0 (8)
C8—C9—H9	120.8	F2A—B1—F1A	113.3 (10)
C8—C9A—C10A	120.9 (11)	F4—B1—F1	109.2 (11)
C8—C9A—H9A	119.5	F3—B1—F1	111.1 (10)
C10A—C9A—H9A	119.5	F4—B1—F2	109.3 (7)
C9A—C10A—C11A	119.3 (13)	F3—B1—F2	104.8 (7)
C9A—C10A—H10A	120.3	F1—B1—F2	109.4 (9)
C11A—C10A—H10A	120.3	F2A—B1—F3A	113.4 (8)
C9—C10—C11	119.0 (9)	F1A—B1—F3A	107.4 (10)
C9—C10—H10	120.5	F2A—B1—F4A	111.2 (7)
C11—C10—H10	120.5	F1A—B1—F4A	104.8 (11)
C12—C11—C10	119.5 (9)	F3A—B1—F4A	106.1 (7)
C12—C11—H11	120.3	F5—B2—F7	119.7 (8)
C10—C11—H11	120.3	F5—B2—F8	112.7 (8)
C12A—C11A—C10A	120.6 (12)	F7—B2—F8	104.0 (6)
C12A—C11A—H11A	119.7	F5A—B2—F7A	117.1 (7)
C10A—C11A—H11A	119.7	F5A—B2—F6A	104.7 (8)
C11—C12—C13	123.3 (11)	F7A—B2—F6A	106.6 (7)
C11—C12—H12	118.4	F5—B2—F6	108.3 (7)
C13—C12—H12	118.4	F7—B2—F6	111.3 (5)
C11A—C12A—C13A	119.1 (14)	F8—B2—F6	98.9 (6)
C11A—C12A—H12A	120.5	F5A—B2—F8A	103.5 (6)
C13A—C12A—H12A	120.5	F7A—B2—F8A	114.9 (4)
C12A—C13A—C8	121.9 (11)	F6A—B2—F8A	109.5 (8)
			
C5—N4—N5—N6	0.3 (2)	C7—C8—C13—C12	179.6 (11)
Co1—N4—N5—N6	−177.23 (13)	C11—C12—C13—C8	−4 (3)
N4—N5—N6—C6	0.1 (2)	C4—N3—C14—C15	83.60 (19)
N4—N5—N6—C7	179.15 (17)	C24—N3—C14—C15	−150.10 (16)
C15—N7—N8—N9	−0.1 (2)	Co1—N3—C14—C15	−32.50 (18)
Co1—N7—N8—N9	179.89 (13)	N8—N7—C15—C16	−0.4 (2)
N7—N8—N9—C16	0.6 (2)	Co1—N7—C15—C16	179.60 (13)
N7—N8—N9—C17	−176.26 (17)	N8—N7—C15—C14	174.10 (17)
C25—N10—N11—N12	0.2 (2)	Co1—N7—C15—C14	−5.9 (2)
Co1—N10—N11—N12	−169.60 (15)	N3—C14—C15—C16	−159.6 (2)
N10—N11—N12—C26	0.2 (3)	N3—C14—C15—N7	27.7 (2)
N10—N11—N12—C27	−178.79 (19)	N8—N9—C16—C15	−0.8 (2)
C3—N2—C1—N1	−0.6 (3)	C17—N9—C16—C15	175.7 (2)
Co1—N2—C1—N1	177.42 (15)	N7—C15—C16—N9	0.7 (2)
C2—N1—C1—N2	0.0 (3)	C14—C15—C16—N9	−172.6 (2)
C1—N1—C2—C3	0.5 (3)	N8—N9—C17—C18	−104.8 (2)
N1—C2—C3—N2	−0.9 (3)	C16—N9—C17—C18	78.9 (3)
C1—N2—C3—C2	0.9 (3)	N9—C17—C18—C23	48.4 (3)
Co1—N2—C3—C2	−176.99 (18)	N9—C17—C18—C19	−135.3 (2)
C14—N3—C4—C5	−154.37 (15)	C23—C18—C19—C20	−0.5 (4)
C24—N3—C4—C5	79.71 (19)	C17—C18—C19—C20	−176.9 (3)
Co1—N3—C4—C5	−37.37 (17)	C18—C19—C20—C21	0.7 (5)
N5—N4—C5—C6	−0.7 (2)	C19—C20—C21—C22	−0.4 (5)
Co1—N4—C5—C6	177.21 (13)	C20—C21—C22—C23	−0.2 (5)
N5—N4—C5—C4	175.96 (17)	C21—C22—C23—C18	0.4 (4)
Co1—N4—C5—C4	−6.2 (2)	C19—C18—C23—C22	0.0 (4)
N3—C4—C5—N4	31.7 (2)	C17—C18—C23—C22	176.3 (2)
N3—C4—C5—C6	−152.8 (2)	C14—N3—C24—C25	81.2 (2)
N5—N6—C6—C5	−0.5 (2)	C4—N3—C24—C25	−152.77 (17)
C7—N6—C6—C5	−179.46 (19)	Co1—N3—C24—C25	−36.46 (19)
N4—C5—C6—N6	0.7 (2)	N11—N10—C25—C26	−0.5 (3)
C4—C5—C6—N6	−175.2 (2)	Co1—N10—C25—C26	170.49 (15)
N5—N6—C7—C8	−101.6 (2)	N11—N10—C25—C24	−179.90 (19)
C6—N6—C7—C8	77.3 (3)	Co1—N10—C25—C24	−8.9 (3)
N6—C7—C8—C9A	91.3 (10)	N3—C24—C25—C26	−146.9 (3)
N6—C7—C8—C13	−74.8 (9)	N3—C24—C25—N10	32.3 (3)
N6—C7—C8—C13A	−87.2 (7)	N11—N12—C26—C25	−0.5 (3)
N6—C7—C8—C9	103.9 (7)	C27—N12—C26—C25	178.4 (2)
C13—C8—C9—C10	1.4 (18)	N10—C25—C26—N12	0.6 (3)
C7—C8—C9—C10	−177.5 (11)	C24—C25—C26—N12	179.8 (2)
C13A—C8—C9A—C10A	−1 (2)	N11—N12—C27—C28	95.8 (3)
C7—C8—C9A—C10A	−179.5 (12)	C26—N12—C27—C28	−83.0 (3)
C8—C9A—C10A—C11A	5 (3)	N12—C27—C28—C33	−14.0 (3)
C8—C9—C10—C11	−1 (2)	N12—C27—C28—C29	168.4 (2)
C9—C10—C11—C12	−3 (2)	C33—C28—C29—C30	−1.1 (4)
C9A—C10A—C11A—C12A	−3 (3)	C27—C28—C29—C30	176.7 (2)
C10—C11—C12—C13	5 (3)	C28—C29—C30—C31	0.6 (4)
C10A—C11A—C12A—C13A	−3 (3)	C29—C30—C31—C32	0.1 (5)
C11A—C12A—C13A—C8	6 (2)	C30—C31—C32—C33	−0.3 (5)
C9A—C8—C13A—C12A	−4.6 (17)	C29—C28—C33—C32	0.9 (4)
C7—C8—C13A—C12A	174.2 (10)	C27—C28—C33—C32	−176.7 (3)
C9—C8—C13—C12	1 (2)	C31—C32—C33—C28	−0.2 (5)
